# Development of a Liquid Chromatography–Tandem Mass Spectrometry (LC–MS/MS) Method for Characterizing Linalool Oral Pharmacokinetics in Humans

**DOI:** 10.3390/molecules28186457

**Published:** 2023-09-06

**Authors:** Yan-Hong Wang, Goutam Mondal, Nicole Stevens, Cécile Bascoul, Russell J. Osguthorpe, Ikhlas A. Khan, Charles R. Yates

**Affiliations:** 1 National Center for Natural Products Research, School of Pharmacy, University of Mississippi, University, MS 38677, USA; 2 doTERRA International, Pleasant Grove, UT 84062, USA; 3 Division of Pharmacognosy, Department of BioMolecular Sciences, School of Pharmacy, University of Mississippi, University, MS 38677, USA

**Keywords:** LC–MS/MS, linalool, human, pharmacokinetics

## Abstract

Lavender (*Lavandula angustifolia* Miller or *Lavandula officinalis* Chaix) is an ethnopharmacological plant commonly known as English lavender. Linalool and linalyl acetate are putative phytoactives in lavender essential oil (LEO) derived from the flower heads. LEO has been used in aroma or massage therapy to reduce sleep disturbance and to mitigate anxiety. Recently, an oral LEO formulation was administered in human clinical trials designed to ascertain its anxiolytic effect. However, human pharmacokinetics and an LC–MS/MS method for the measurement of linalool are lacking. To address this deficiency, a rapid and sensitive liquid chromatography–tandem mass spectrometry (LC–MS/MS) method was developed for the analysis of linalool in human serum. Prior to the analysis, a simple sample preparation protocol including protein precipitation and liquid–liquid extraction of serum samples was created. The prepared samples were analyzed using a C18 reversed-phase column and gradient elution (acetonitrile and water, both containing 0.1% formic acid). A Waters Xevo TQ-S tandem mass spectrometer (positive mode) was used to quantitatively determine linalool and IS according to transitions of *m/z* 137.1→95.1 (t_R_ 0.79 min) and 205.2→149.1 (t_R_ 1.56 min), respectively. The method was validated for precision, accuracy, selectivity, linearity, sensitivity, matrix effects, and stability, and it was successfully applied to characterize the oral pharmacokinetics of linalool in humans. The newly developed LC–MS/MS-based method and its application in clinical trial serum samples are essential for the characterization of potential pharmacokinetic and pharmacodynamic interactions.

## 1. Introduction

Lavender (*Lavandula angustifolia* Miller or *Lavandula officinalis* Chaix) commonly known as English lavender has a long ethnopharmacologic history. The essential oil obtained from lavender is produced by steam distillation of the foliage and flower heads, which results in a more aromatic oil compared to the foliage alone [1]. The main phytochemical actives found in lavender essential oil derived from the flower heads include linalool, linalyl acetate, lavandulyl acetate, terpinen-4-ol, β-ocimene (*cis*- and *trans*-), and 1,8-cineole. *L. angustifolia* is relatively low in camphor, but contains higher levels of terpenes (e.g., β-phellandrene) and sesquiterpenes (e.g., caryophyllene) [2]. Consequently, *L. angustifolia* has historically found use in perfumes and cosmetics as opposed to high camphor species (e.g., *L. stoechas*), which have been employed as insect repellants.

Historically, lavender essential oil (LEO) has been most frequently used in aroma or massage therapy to reduce sleep disturbance and to mitigate anxiety. A recent meta-analysis of clinical studies designed to investigate the therapeutic effect of lavender oil in patients with subsyndromal anxiety or generalized anxiety disorder found that lavender oil exerted an anxiolytic effect and had beneficial effects on anxiety co-morbidities such as disturbed sleep and decreased quality of life [3]. The neuropharmacological effects of LEO have been linked to the monoterpenes linalool and linalyl acetate, which are ligands for voltage-dependent calcium channels and the NMDA receptor [4,5]. In preclinical studies, inhalation of linalool reduces anxiolytic activity in the rodent light/dark, aggressive behavior, and social interaction models [6,7,8]. Recently, an oral LEO formulation, containing 36.8% linalool and 34.2% linalyl acetate [5], was administered in human clinical trials designed to ascertain its anxiolytic effect [9]. Despite the aforementioned clinical pharmacology studies, critical information is lacking regarding the oral pharmacokinetics of LEO phytoactives. The lack of simple, robust methods for detection of linalool in plasma has contributed to this gap in linalool pharmacology.

In earlier studies, linalool and LEO were mainly analyzed using GC–FID and GC–MS methods on the basis of their volatile properties [10,11,12,13,14,15,16,17]. Most of these reported methods suffer from long GC run times and high detection limits, requiring large quantities of blood and serum, or complicated methods for biological sample preparation. Famiglini et al. reported an LC–MS method for the analysis of linalool in personal care products using a nano-column [18]. However, this method is unsuitable for the bioanalysis of blood or serum samples because of the long run time, high detection limits, and baseline interference. In recent years, liquid chromatography–tandem mass spectrometry (LC–MS/MS) techniques have been successfully applied for bioanalytical analysis due to the high selectivity and sensitivity of this technique [19]. To address the need for robust methods for linalool detection, this study aimed to develop and validate a rapid, simple, and sensitive liquid chromatography–tandem mass spectrometry (LC–MS/MS) method for the determination of linalool in human serum and to characterize its pharmacokinetics after oral administration to humans.

## 2. Results and Discussion

### 2.1. Method Development and Qualification

*trans*, *trans*-Farnesol was selected as the internal standard (IS) due to its resemblance chemical structure, physicochemical property, and mass spectrometric characteristics to those of linalool. The mobile phase composition, retention time, flow rate, and suitable chromatographic column were assessed to optimize chromatographic settings. The UPLC BEH C18 column (50 mm × 2.1 mm I.D., 1.8 µm) achieved baseline separation for linalool (t_R_ 0.79 min) and *trans*, *trans*-farnesol (t_R_ 1.56 min) within a two-minute runtime, which provided satisfactory results in terms of shorter runtime, analyte separation, and peak shape. An acetonitrile-based mobile phase had lower background noise and system pressure than methanol. The addition of 0.1% formic acid greatly enhanced the intensities of the peaks. Optimal chromatographic separation was observed using a solvent composition of acetonitrile with 0.1% formic acid (*v*/*v*) and water containing 0.1% formic acid as the mobile phase. It was also observed that gradient elution at a flow rate of 0.37 mL/min, eluted as 0–1.6 min, 60% B to 78% B, and 1.6–2.0 min, 78% B to 100% B, significantly improved response intensity, resolution, and peak shape.

**Figure 1:**
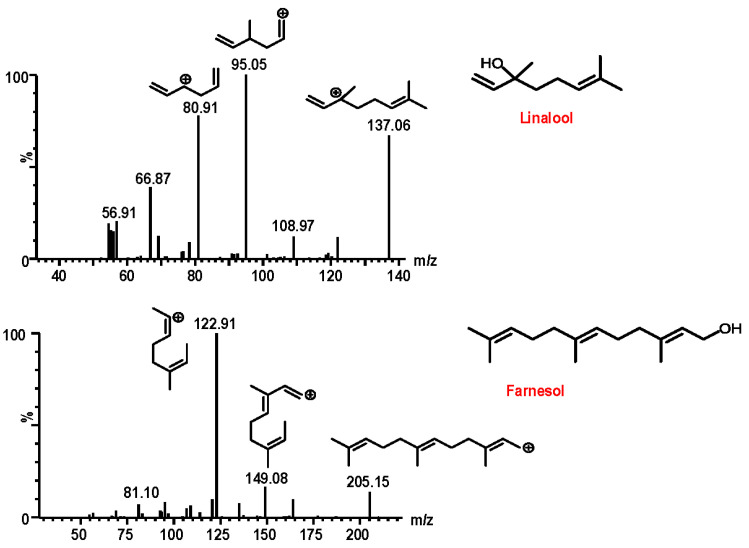
MS/MS spectra of linalool and *trans*, *tarns*-farnesol.

In order to achieve maximal sensitivity for analytes linalool and IS, tandem mass parameters including targeted ion selection, capillary voltage, cone voltages, desolvation temperature, desolvation gas flow rate, and collision energy were optimized in ESI positive ionization modes for each analyte using a 1 µg/mL tuning solution in acetonitrile. The protonated ions of linalool were barely detected at a 150 °C source temperature. However, while optimizing desolvation temperature at 300 °C and desolvation gas flow rate as 800 L/h, steady product ions of linalool were found at *m/z* 137, which further yielded an ion at *m/z* 95 (Figure 1). Under optimized conditions, protonated ions of *trans*, *trans*-farnesol were detected at *m/z* 205, which generated product ions at *m/z* 149 and 123. Two MRMs were chosen to be quantifier and qualifier for each analyte. The most intense peaks, which were used for analyte quantification, were observed at *m/z* 95.1 for linalool and 149.1 for IS. The MS parameters of quantifier and qualifier ions such as cone voltage and collision energy for all analytes are listed in Table 1.

A simple protein precipitation sample preparation protocol was selected due to its wide use in LC/MS sample preparation for bioanalysis, as well as its simplicity and low cost. Optimization of the method was initially focused on the choice of a suitable organic extraction solvent (methanol, acetonitrile, and isopropanol). Acetonitrile provided the best analyte recovery and peak shape compared with those of methanol and isopropanol.

### 2.2. Method Validation

Selectivity, linearity, sensitivity, precision, accuracy, recovery, matrix effects, and stability were evaluated for validation of developed method.

#### 2.2.1. Selectivity

**Figure 2:**
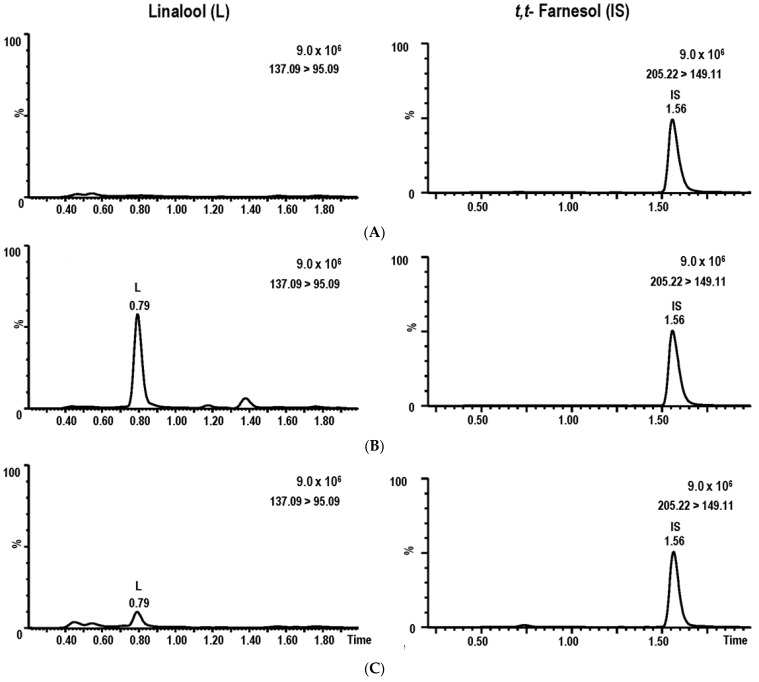
MRM chromatograms of linalool (L) and *trans*, *tarns*-farnesol (IS) in drug-free serum spiked with IS (**A**), a serum QC sample (**B**), and a serum sample collected at 1.5 h (**C**).

Assay selectivity was evaluated at the LOQ using drug-free human serum samples from different serum lots. No interfering peaks at the retention times of linalool (t_R_ 0.79 min) and IS (t_R_ 1.56 min) were observed in blank serum. Representative chromatograms of drug-free human serum spiked with 500 ng/mL IS, a serum QC sample (250 ng/mL linalool with 500 ng/mL IS), and a serum sample collected at 1.5 h after administration of linalool are shown in Figure 2.

#### 2.2.2. Linearity and Sensitivity

To minimize matrix effects, standard solutions containing IS were spiked into the drug-free human serum and extracted using the optimized method. The calibration curve of linalool was Y = 0.8466X + 12.36, which was established by plotting the mean peak response of analyte/IS versus the corresponding known concentration of linalool. The linear range of the linalool calibration curve was over 7.5–500 ng/mL in human serum with a correlation coefficient of determination (r2) greater than 0.99 (*n* = 14). The limit of detection (LOD) and limit of quantification (LOQ) were defined as a signal-to-noise ratio of 3 and 10, respectively, corresponding to 3.5 and 7.5 ng/mL in human serum.

#### 2.2.3. Precision and Accuracy

Intra- and inter-day precision and accuracy of the developed method were evaluated by analyzing four (*n* = 4) QC samples at two concentrations (50 and 250 ng/mL) on the same day and three different days, respectively. As shown in Table 2, both intra-day and inter-day precision and accuracy values in serum were well within the 20% acceptance range. The relative standard derivation (RSD, %) for intra- and inter-day precision values in serum were below 5%, and the accuracies of intra- and inter-day assays in serum were 97.1–99.3%.

#### 2.2.4. Stability

Stock solutions of linalool and *trans*, *trans*-farnesol in acetonitrile solvent stored at −20 °C were stable for up to three months without any changes in peak areas or the appearance of any extra peaks. The stability of short-term storage and post-treatment storage for linalool in human serum showed no significant degradation, and the variations of all testing samples were within ±15% deviation between the predicted and nominal concentrations.

### 2.3. Pharmacokinetics

**Figure 3:**
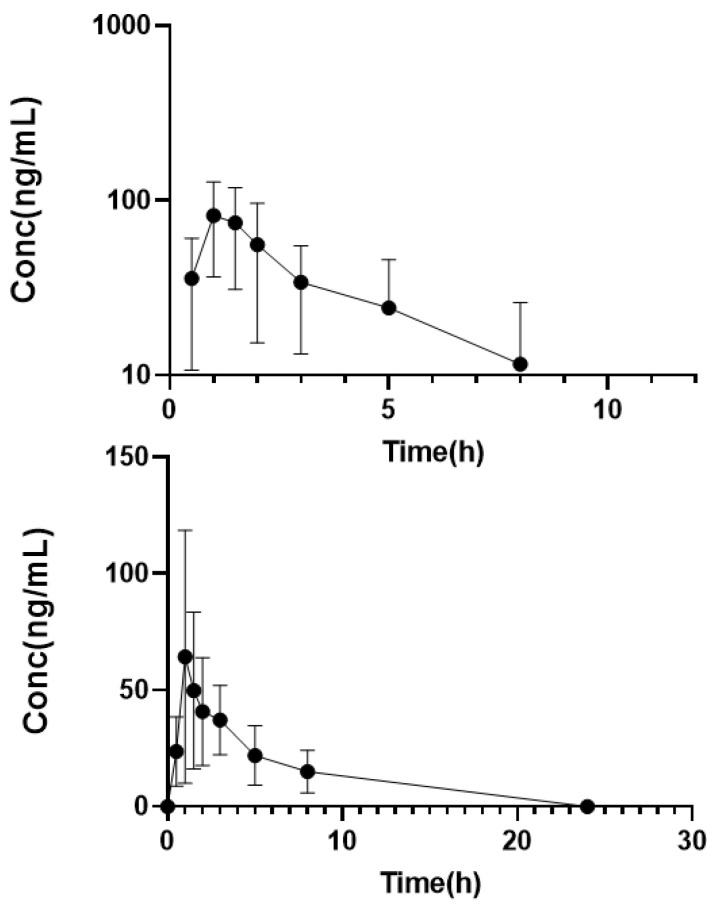
Serum concentration versus time profile for linalool. Subjects received an oral dose of linalool (100 mg). Data represent the mean ± SD.

The aforementioned validated LC–MS/MS method was used to characterize linalool oral pharmacokinetics in humans. Serum concentration (mean ± standard deviation) time profiles for linalool are depicted in Figure 3. Individual linalool pharmacokinetic parameters are listed in Table 3. Linalool was rapidly absorbed following oral administration reaching a mean maximum concentration (C_max_) of 85.5 ± 42.6 ng/mL approximately one hour (T_max_) after ingestion. Oral clearance (CL/F) and volume of distribution (V_d_/F) were 385 ± 287 L/h and 1495 ± 898.4 L, respectively, with an associated half-life of 3.9 (±2.9) h. Linalool plasma concentrations were detected up to eight hours post dosing with serum AUC values at 442 ± 243 h·ng/mL.

In the natural complex substance LEO, linalyl acetate can be viewed as a pro-drug for linalool. Thus, in order to determine the contribution of linalyl acetate to linalool serum exposure, we administered linalyl acetate separately and measured serum linalool concentrations. At the linalyl acetate dose tested, linalool serum levels were found to be less than LLOQ for the majority of samples. Consequently, linalool exposure following linalyl acetate administration represented approximately one-tenth that following linalool administration (64.9 ± 78.0 vs. 442 ± 243 h·ng/mL).

## 3. Materials and Methods

### 3.1. Reagents and Chemicals

Linalool (≥97%), *trans*, *trans*-farnesol (96%), and human serum free of linalool and *trans*, *trans*-farnesol were purchased from Sigma–Aldrich (St. Louis, MO, USA). HPLC-grade acetonitrile, methanol, and formic acid were purchased from Fisher Scientific (Fair Lawn, NJ, USA). Water used for the HPLC mobile phase was purified using a Millipore Synergy UV Water Purification System (Millipore SAS, Molsheim, France).

### 3.2. Preparation of Calibration Standards and Quality Controls

Stock solutions of linalool (L) and *trans*, *trans*-farnesol (F, Internal Standard, IS) were prepared in methanol (1.0 mg/mL).) Working solutions of each compound were prepared from stock solutions. For preparing calibration standard of linalool, an aliquot (100 µL) of standard solution containing 15 ng/mL of L and 1000 ng/mL of F was added to drug-free human serum (100 µL) to a final concentration of L (7.5 ng/mL) and F (500 ng/mL). Serum containing L and F was mixed by vortexing for 5 min, and the mixture was then centrifuged (12,000× *g* for 10 min at 4 °C). The supernatant was then transferred to another tube. Similarly, different concentrations of linalool were prepared at 7.5, 10, 25, 50, 100, 250, and 500 ng/mL. Quality control (QC) samples were added working solution of standards to drug-free human serum at final concentrations of 50 and 250 ng/mL, respectively. The final concentration of IS was 500 ng/mL for routine use in all prepared solutions. The calibration curve for linalool in human serum was derived from the peak area ratios relative to that of *trans*, *trans*-farnesol from the linear regression with weighting factor of 1/x. The QC samples were analyzed along with each batch of serum samples to assess the intra- and inter-day precision and accuracy of the method. All prepared solutions were stored at 4 °C prior to LC–MS/MS analysis.

### 3.3. Sample Preparation

Protein precipitation was used to extract linalool from human serum. Acetonitrile (ACN) solvent (100 μL) containing IS (1000 ng/mL) was added to human serum (100 μL) in a 1.5 mL micro-centrifuge tube and vortexed for 5 min. The mixture was then centrifuged at 12,000× *g* for 10 min at 4 °C and the supernatant was transferred to an LC vial for LC–MS/MS analysis. All prepared samples were stored at 4 °C prior to LC–MS/MS analysis.

### 3.4. LC–MS/MS Parameters

The LC–MS/MS system comprised a Waters Acquity UPLC^TM^ I-class system (Waters Corp., Milford, MA, USA), equipped with a binary solvent manager, sample manager, heated column compartment, and Xevo TQ-S triple-quadrupole mass spectrometry detector. The instrument was controlled by Waters MassLynx 4.1 software. Chromatographic separation of linalool was carried out using a Waters UPLC BEH C18 column (50 mm × 2.1 mm I.D., 1.8 µm), maintained at 40 °C and 10 °C for the column and sample temperature, respectively. The mobile phase consisted of (A) water containing 0.1% formic acid and (B) acetonitrile with 0.1% formic acid. The gradient elution was applied for analysis at a flow rate of 0.37 mL/min and programmed as follows: 0–1.6 min, 60% B to 78% B; 1.6–2.0 min, 78% B to 100% B. The analysis was followed by a one-and-a-half minute washing procedure with 100% B and re-equilibration period of 3.5 min with initial condition. A wash solvent (1:1:1:1 methanol/acetonitrile/isopropanol/ water, *v*/*v*) and purge solvent (1:1 methanol/water, *v*/*v*) were used for the autosampler and needle wash. The injection volume was 10 µL.

MS/MS analysis was performed on a Waters Xevo TQ-S triple-quadrupole mass spectrometer (Waters Corp., Milford, MA, USA) that was connected to the UHPLC system via an electrospray ionization (ESI) interface. The ESI MS/MS parameters were set as follows: capillary voltage, 4.0 kV; source temperature, 150 °C; desolvation temperature, 300 °C; desolvation gas flow, 800 L/h; cone gas flow, 150 L/h. Nitrogen was used as the desolvation and cone gas. Argon (99.99% purity) was introduced as the collision gas into the collision cell at a flow rate of 0.15 mL/min. The effluent was introduced into the TQ-S mass spectrometer equipped with ESI in positive ion mode (ESI+) for quantification of the analytes. Detection was obtained by multiple reaction monitoring (MRM) mode including two MRMs for confirmation of the analytes. The quantification of linalool was acquired with transitions of key product ions at *m/z* 137.1→95.1 (dwell time 24 ms, cone voltage 39 V, and collision energy 10 eV) and protonated ion of *trans*, *trans*-farnesol at *m/z* 205.2→149.1 (dwell time 24 ms, cone voltage 10 V, and collision energy 10 eV). Data acquisition was carried out using the MassLynx 4.1 software (Waters Corp., Milford, MA, USA).

### 3.5. LC–MS/MS Method Validation

Method validation was performed in accordance with the criteria suggested by the US Food and Drug Administration (FDA) Guidance for Industry-Bioanalytical Method Validation 19. Method parameters such as specificity, linearity, sensitivity, precision, accuracy, and stability of linalool were validated in human serum. Method specificity was evaluated by comparing chromatograms of nine drug-free human blank serums for interference at the retention times of linalool and *trans*, *trans*-farnesol (IS).

Calibration curves in human serum were constructed by plotting the peak ratios of linalool to the IS against the nominal concentrations of the calibration standards at 7.5, 10, 25, 50, 100, 250, and 500 ng/mL. Duplicate measurements of seven concentrations of linalool were used to construct the calibration curve for each sample batch. The linear least-squares regression of the calibration lines, slopes, intercepts, and correlation coefficients were obtained from the peak area ratios of linalool to IS versus corresponding concentrations. Unknown sample concentrations of linalool were calculated from the linear regression with weighting factor of 1/x. The limit of detection (LOD) and limit of quantification (LOQ) were defined as the lowest concentration with signal-to-noise ratios of 3 and 10, respectively. For LOQ determination, the acceptable accuracies of 80–120% and sufficient precisions within 20% were adopted and verified using seven replicate analyses.

QC samples at two different concentrations (50 and 250 ng/mL) were used to evaluate the intra-day precision and accuracy on the same day and inter-day precision and accuracy on three different days. Each batch of analysis consisted of a serum blank, seven concentrations of calibration standards, and different QC samples at two concentrations. Precision was expressed as the relative standard deviation (RSD, %), and accuracy was expressed as [(mean detected concentration)/(nominal concentration) × 100%]. The stability of linalool in serum was assessed by analyzing four replicate samples (50 and 250 ng/mL) under short-term storage (8 h at room temperature) and post-treatment storage (72 h at 4 °C). The peak areas of the linalool and IS obtained from freshly prepared samples were considered as the reference to measure the relative stability at short-term and long-term points.

### 3.6. Pharmacokinetic Study Design

A single-center, randomized, double-blind study was conducted in human volunteers to determine the oral pharmacokinetics of linalool. The data presented herein represent two of the study’s arms, viz., linalool and linalyl acetate oral administration. Review and approval of the clinical study protocol and informed consent were conducted by Solutions Institutional Review Board (IRB number 2020/01/14). Consistent with the Declaration of Helsinki (2008), the study followed Good Clinical Practice as set forth in the 1996 guidelines of the International Conference on Harmonization. In addition, the study complied with federal regulations that included the US Code of Federal Regulations (CFR) governing Protection of Human Subjects (21 CFR Part 50), Institutional Review Board (IRB) (21 CFR Part 56), and Financial Disclosure by Clinical Investigator (21 CFR Part 54).

Once informed consent was obtained, study participants (23 female, 16 male, ages 19–64 inclusive) received an oral dose of either linalool or linalyl acetate administered as two hypromellose (hydroxypropyl methylcellulose) capsules each containing either 50 mg ± 5% linalool and 150 mg ± 5% olive oil or 45 mg ± 5% linalyl acetate and 150 mg ± 5% olive oil. These doses reflect the constituent ratios in the lavender essential oil used for the pharmacokinetic study. Serial venous blood samples were obtained before dosing (0) and 0.5, 1.0, 1.5, 2.0, 3.0, 5.0, 8.0, and 24 h post dosing. Blood samples were centrifuged within 30 min of collection (3400 rpm for 15 min). Serum was removed and stored at −80 °C until analysis by LC–MS/MS.

### 3.7. Pharmacokinetic Data Analysis

Noncompartmental pharmacokinetic analyses of the linalool concentrations were analyzed using Phoenix WinNonlin (Version 7.0; Pharsight Corporation, Mountain View, CA, USA) with adjustment for lag time after oral administration. The maximum concentration (C_max_) and time corresponding to the C_max_ (T_max_) were determined from the serum concentration versus time data. The area under the serum concentration–time curve from time 0 to infinity (AUC0–∞) was determined using the linear trapezoidal rule. The terminal half-life (t_1/2_) was calculated using ln 2/k_el_, with k_el_ as the terminal rate elimination constant estimated from the slope of the linear regression of the log serum concentration versus the time curve during the terminal phase. Oral clearance (CL/F) was calculated by dividing the oral dose by AUC_0_–∞, and the oral volume of distribution (V_d_/F) during the terminal elimination phase was calculated by dividing CL/F by k_el_.

## 4. Conclusions

Lavender essential oil (LEO), containing linalool as its principal active ingredient, has demonstrated neuropharmacological effects in both animal models and humans [2,4,5,6,7,8,9]. However, there is a lack of studies investigating the pharmacokinetics of LEO or linalool in humans. In this research, we aimed to address this gap by developing and validating a highly sensitive LC–MS/MS-based bioanalytical method to measure linalool concentrations in serum. Subsequently, we utilized this method to examine the oral pharmacokinetics of linalool in humans. The key findings from our study are as follows: (1) the developed method exhibited robustness and was capable of accurately quantifying linalool levels in the low ng/mL range, comparable to previous GC-based methods; (2) the method met the required criteria for characterizing the oral pharmacokinetics of linalool in humans, establishing its suitability for this purpose; (3) linalool was rapidly absorbed following oral administration, reaching peak concentration approximately one hour after administration.

In previous studies, in both humans and animals, linalool levels were quantified in various matrices such as blood and exhaled air using gas chromatography (GC)-based methods [10,11,12,13,14,15,16,17]. However, these methods have certain limitations, including long run times, poor sensitivity, the requirement for large sample volumes, and labor-intensive sample preparation procedures. Famiglini et al. reported an LC–MS method for analyzing linalool in personal care products using a nano-column [18]. Nevertheless, this particular method is not suitable for the bioanalysis of blood or serum samples due to its lengthy run time, high detection limits, and baseline interference. The method developed and employed in this study overcomes these limitations by demonstrating enhanced sensitivity, shorter run times, and simplified sample preparation.

In a previous pharmacokinetic study conducted in rats, the oral administration of equimolar doses of linalool (28.9 mg/kg), linalyl acetate (36.8 mg/kg), or LEO (100 mg/kg) resulted in maximum linalool concentrations of 33, 10, and 77 ng/mL, respectively. These findings indicated that the relative oral bioavailability of linalool was higher when administered as the total oil compared to linalool or linalyl acetate alone [20]. Subsequent repeat dose experiments in rats, involving oral administration of LEO for 14 consecutive days, revealed that linalool did not accumulate in the plasma. This suggests that linalool is effectively eliminated within a single 24-h dosing interval [21].

Several attempts have been made to enhance the oral bioavailability of linalool. For instance, the preparation of linalool-loaded nanostructured lipid or beta-cyclodextrin carriers significantly improved its oral bioavailability in rats [22,23]. In our study, an oral linalool dose of 100 mg resulted in a maximum concentration of 85 ng/mL (Figure 3). According to the aforementioned study [20], an estimated linalool dose of approximately 75 mg can be inferred (28.9 mg/kg, assuming an average weight of 0.4 kg for rats). Thus, it appears that linalool oral bioavailability is slightly higher in humans (100 mg dose, 85 ng/mL Cmax) compared to rats (75 mg dose, 33 ng/mL Cmax). Plasma linalool levels following oral administration of linalyl acetate were approximately one-tenth of those observed after oral administration of linalool, indicating that linalyl acetate contributes minimally to the linalool plasma area under the curve. This finding aligns with the previous study [20]. Future studies will examine linalool pharmacokinetics in humans following oral administration of LEO to determine if linalool oral bioavailability is improved as reported in rats. Future investigations will focus on exploring linalool pharmacokinetics in humans following oral administration of LEO. These studies aim to determine whether linalool oral bioavailability is improved as reported in rats.

## Figures and Tables

**Table 1:** Settings of MS parameters for the quantification of linalool and IS.

Compound Name	Target Ions (*m/z*)	Quantifier			Qualifier		
		Ions (*m/z*)	Conne (V)	Collision Energy (eV)	Ions (*m/z*)	Conne (V)	Collision Energy (eV)
Linalool	137.1	95.1	39.0	10.0	81.0	39.0	10.0
IS	205.2	149.1	10.0	10.0	123.0	10.0	14.0

**Table 2:** The precision and accuracy of intra- and inter-day assays of serum samples.

Analyte	Nominal Conc. (ng/mL)	Intra-Day									Inter-Day		
		Day-1			Day-2			Day-3					
		Dect. Conc. (ng/mL)	RSD (%)	Accu. (%)	Dect. Conc. (ng/mL)	RSD (%)	Accu. (%)	Dect. Conc. (ng/mL)	RSD (%)	Accu. (%)	Dect. Conc. (ng/mL)	RSD (%)	Accu. (%)
Linalool	50	49.49	0.17	98.9	48.56	0.37	97.1	49.12	0.66	98.2	49.06	0.91	98.1
	250	248.23	0.12	99.3	248.14	0.17	99.3	247.88	0.35	99.2	248.09	0.22	99.2

Dect. Conc. = detected concentration; RSD = relative standard derivation of precision values; Accu. = accuracy.

**Table 3:** Linalool pharmacokinetic parameters.

PK Parameters of Linalool	Treatment Allocation
	Linalool (*n* = 10)
C_max_ (ng/mL)	85.49 ± 42.56
T_max_ (h)	1.15 ± 0.24
t_1/2_ (h)	3.93 ± 2.94
AUC (h·ng/mL)	441.86 ± 242.69
CL/F (L/h)	384.83 ± 287.04
V_d_/F (L)	1495.67 ± 898.38

## Data Availability

The data presented in this study are available on request from the corresponding author.

## Sample Availability

Not available.
